# Automated mitotic spindle hotspot counts are highly associated with clinical outcomes in systemically untreated early-stage triple-negative breast cancer

**DOI:** 10.1038/s41523-024-00629-3

**Published:** 2024-03-29

**Authors:** Roberto A. Leon-Ferre, Jodi M. Carter, David Zahrieh, Jason P. Sinnwell, Roberto Salgado, Vera J. Suman, David W. Hillman, Judy C. Boughey, Krishna R. Kalari, Fergus J. Couch, James N. Ingle, Maschenka Balkenhol, Francesco Ciompi, Jeroen van der Laak, Matthew P. Goetz

**Affiliations:** 1 Mayo Clinic, Rochester, MN USA; 2 University of Alberta, Edmonton, Canada; 3 GZA-ZNA-Hospitals, Antwerp, Belgium; 4 Peter Mac Callum Cancer Centre, Melbourne, Australia; 5 Radboud University Medical Center, Nijmegen, Netherlands; 6 Center for Medical Image Science and Visualization, Linköping University, Linköping, Sweden

**Keywords:** Prognostic markers, Breast cancer

## Abstract

Operable triple-negative breast cancer (TNBC) has a higher risk of recurrence and death compared to other subtypes. Tumor size and nodal status are the primary clinical factors used to guide systemic treatment, while biomarkers of proliferation have not demonstrated value. Recent studies suggest that subsets of TNBC have a favorable prognosis, even without systemic therapy. We evaluated the association of fully automated mitotic spindle hotspot (AMSH) counts with recurrence-free (RFS) and overall survival (OS) in two separate cohorts of patients with early-stage TNBC who did not receive systemic therapy. AMSH counts were obtained from areas with the highest mitotic density in digitized whole slide images processed with a convolutional neural network trained to detect mitoses. In 140 patients from the Mayo Clinic TNBC cohort, AMSH counts were significantly associated with RFS and OS in a multivariable model controlling for nodal status, tumor size, and tumor-infiltrating lymphocytes (TILs) (*p* < 0.0001). For every 10-point increase in AMSH counts, there was a 16% increase in the risk of an RFS event (HR 1.16, 95% CI 1.08–1.25), and a 7% increase in the risk of death (HR 1.07, 95% CI 1.00–1.14). We corroborated these findings in a separate cohort of systemically untreated TNBC patients from Radboud UMC in the Netherlands. Our findings suggest that AMSH counts offer valuable prognostic information in patients with early-stage TNBC who did not receive systemic therapy, independent of tumor size, nodal status, and TILs. If further validated, AMSH counts could help inform future systemic therapy de-escalation strategies.

## Introduction

Compared to patients with hormone receptor (HR)-positive or HER2-amplified tumors, patients with operable triple-negative breast cancer (TNBC) have a higher risk of early recurrence and death^1,2^. Given this, most patients with operable TNBC are recommended to receive adjuvant or neoadjuvant multiagent systemic therapy. However, beyond patient age, tumor, and nodal status, no other clinicopathologic factors or biomarkers are used in the clinic to refine prognosis estimation based on disease biology or to guide the use of—or intensity of—systemic therapy.

Most TNBC tumors are of high histologic grade, highly proliferative, and characterized by abundant mitoses. As such, classic histological grade determination, manual mitosis counting, or proliferation biomarkers (e.g., Ki-67, mitotic activity index, among others) have not offered meaningful prognostic value in the clinic when evaluating unselected cohorts of early-stage TNBC^3–5^. While artificial intelligence (AI)-based automated mitosis counting tools have been developed and correlate well with manual mitosis counting, these tools have also failed to identify any prognostic value in patients with TNBC treated with adjuvant chemotherapy^6–11^. However, no studies have evaluated the prognostic effects of such biomarkers in systemically untreated patients.

Recently, it has been identified that high levels of tumor-infiltrating lymphocytes (TILs) are highly prognostic in early-stage TNBC, even in the absence of adjuvant or neoadjuvant systemic therapy^4,12–14^. Patients with stage I TNBC and TILs ≥50% treated exclusively with locoregional therapy exhibited 5-year recurrence-free survival (RFS) and overall survival (OS) rates exceeding 90%^14^. While proliferation biomarkers have historically not been prognostic in TNBC^5^, these evaluations have not included significant numbers of patients who did not receive systemic therapy. Given that chemotherapy may have a differential impact on high versus low proliferating tumors, the prognostic value of proliferation biomarkers may be obscured when being evaluated in cohorts of patients treated with chemotherapy. Identifying prognostic biomarkers that can recapitulate the natural history of early-stage TNBC in the absence of systemic therapy and help identify patients at the lowest versus highest risk of recurrence or death is critical to inform future systemic therapy optimization strategies. Here, we evaluated a strategy using fully automated mitotic spindle hotspot (AMSH) counting to determine its association with RFS and OS in two independent cohorts of early-stage TNBC not treated with systemic therapy.

## Results

### Patient characteristics

A total of 182 patients in the Mayo cohort and 130 patients in the Radboud cohort were treated with locoregional therapy but no adjuvant or neoadjuvant systemic therapy. AMSH counts could be obtained in tumors from 140 patients from the Mayo Cohort (29 had no available digitized slides, and 13 had minimal/no tumor present on digitized slide). Four patients from the Radboud cohort were excluded from analysis due to having adenoid cystic carcinoma (an indolent subtype of TNBC also previously excluded from the Mayo Cohort)^4^, leaving 126 evaluable patients for the Radboud Cohort.

Clinicopathological variables, including tumor size, nodal status, histological grade, and stromal TILs were available for most patients in both cohorts. Ki-67 and menopausal status were only available in the Mayo cohort. The baseline characteristics of both cohorts are shown in Table 1. Patients were most often older than 55, with tumors measuring ≤2 cm, lymph node-negative, and high-grade histology. Most tumors had <30% TILs. Baseline characteristics were similar between the two cohorts, except for a higher proportion of younger patients and lower TIL levels in the Mayo Cohort.

**Table 1:** Characteristics of the study population at baseline

Characteristic	Mayo cohort *N* = 140	Radboud cohort *N* = 126	*P*
Age group, *n* (%)			
<55 years	48 (34%)	19 (15%)	<0.001
≥55 years	92 (66%)	107 (85%)	
Menopausal status, *n* (%)			
Premenopausal	41 (29%)	Not Available	-
Postmenopausal	99 (71%)		
Tumor size, *n* (%)			
≤2 cm	94 (67%)	76 (60%)	0.247
>2 cm	46 (33%)	50 (40%)	
Nodal status, *n* (%)			
Negative	116 (87%)	102 (81%)	0.167
Positive	17 (13%)	24 (19%)	
Missing	7	0	
Histologic grade, *n* (%)			
Grade 1	4 (3%)	1 (1%)	0.419
Grade 2	18 (13%)	19 (15%)	
Grade 3	118 (84%)	106 (84%)	
Stromal TILs, *n* (%)			
≥30	50 (36%)	62 (53%)	0.006
<30	89 (64%)	55 (47%)	
Missing	1	9	
Stromal TILs, *n* (%)			
≥50	28 (20%)	35 (30%)	0.071
<50	111 (80%)	82 (70%)	
Missing	1	9	
Ki-67 proliferative index, *n* (%)			
≤15%	36 (26%)	Not Available	-
>15%	102 (74%)		
Missing	2		
AMSH counts			
Mean (SD)	32.2 (34.3)	44.6 (35.1)	0.004
Median (Q1, Q3)	18.0 (8.0, 42.2)	36 (16.5, 65.2)	
Minimum, maximum	0, 211	1, 164	

*SD* standard deviation, *Q1* first quartile, *Q3* third quartile, *TILs* tumor-infiltrating lymphocytes, *AMSH* automated mitotic spindle hotspot.

*P* values are two-sided and based on the Chi-square test for categorical variables and the two-sample *t* test for continuous variables.

### Automated mitotic spindle hotspot counts and associations with clinicopathological characteristics and molecular subtype

The mean and median AMSH counts for both cohorts are shown in Table 1. AMSH counts were lower in the Mayo Cohort (median [Q1, Q3]: 18.0 [8.0, 42.2]) than in the Radboud cohort (median [Q1, Q3]: 36, [16.5, 65.2]). AMSH count distribution for both cohorts is shown in Supplementary Figure 1. We did not observe evidence of AMSH drift over time from the date of breast cancer surgery in either cohort (Supplementary Figure 1, panels C and D).

We evaluated whether the AMSH counts were associated with relevant clinicopathologic factors (Table 2). As expected, we observed that higher AMSH counts categorized as terciles were linearly associated with histological grade and Ki-67. Lower AMSH counts were seen in older patients in the Mayo Cohort, but not in the Radboud cohort. Tumors with lower AMSH counts were smaller (more often measuring ≤2 cm) and node-negative. AMSH counts did not differ according to stromal TILs in the Mayo Cohort but were higher among patients with TIL-rich tumors in the Radboud cohort. Molecularly defined Luminal Androgen Receptor (LAR) TNBC tumors (determined by RNA seq) in the Mayo Cohort^15^ had lower AMSH counts than non-LAR TNBC tumors. The molecular subtype was not available in the Radboud Cohort.

**Table 2:** Cross-tabular summary of each binary factor and ordered AMSH count by cohort

Binary factor	Mayo cohort mitotic spindle hot spot terciles			*P*	Radboud cohort (thresholds based on the Mayo cohort)			*P*
	0–11 (*N* = 44)	12–34 (*N* = 49)	≥35 (*N* = 47)		0–11 (*N* = 23)	12–34 (*N* = 38)	≥35 (*N* = 65)	
Age group								
<55 years	9 (21%)	16 (33%)	23 (49%)	0.004	4 (17%)	6 (16%)	9 (14%)	0.665
≥55 years	35 (80%)	33 (67%)	24 (51%)		19 (83%)	32 (84%)	56 (86%)	
Menopausal status								
Premenopausal	8 (18%)	14 (29%)	19 (40%)	0.020	Not available			
Postmenopausal	36 (82%)	35 (71%)	28 (60%)					
Tumor size								
≤2 cm	37 (84%)	34 (69%)	23 (49%)	<0.001	20 (87%)	26 (68%)	30 (46%)	<0.001
>2 cm	7 (16%)	15 (31%)	24 (51%)		3 (13%)	12 (32%)	35 (54%)	
Nodal status								
Negative	40 (95%)	41 (89%)	35 (78%)	0.014	22 (96%)	31 (82%)	49 (75%)	0.038
Positive	2 (5%)	5 (11%)	10 (22%)		1 (4%)	7 (18%)	16 (25%)	
Histologic grade								
Grade 1–2	14 (32%)	7 (14%)	1 (2%)	<0.001	16 (70%)	4 (11%)	0 (0%)	<0.001
Grade 3	30 (68%)	42 (86%)	46 (98%)		7 (30%)	34 (90%)	65 (100%)	
Stromal TILs								
≥30	13 (30%)	19 (39%)	18 (38%)	0.434	4 (21%)	20 (59%)	38 (59%)	0.012
<30	30 (70%)	30 (61%)	29 (62%)		15 (79%)	14 (41%)	26(41%)	
Stromal TILs								
≥50	6 (14%)	13 (27%)	9 (19%)	0.564	0 (0%)	13 (38%)	22 (34%)	0.021
<560	37 (86%)	36 (74%)	38 (81%)		19 (100%)	21 (62%)	42 (66%)	
Ki-67 proliferative index								
≤15%	22 (51%)	10 (20%)	4 (9%)	<0.001	Not available			
>15%	21 (49%)	39 (80%)	42 (91%)					
LAR subtype								
Non-LAR	5 (50%)	15 (63%)	31 (89%)	0.004	Not available			
LAR	5 (50%)	9 (38%)	4 (11%)					

*P* values are two-sided and based on the Cochrane-Armitage test for trend. Column percentages are shown and may not add to 100% due to rounding. In the Mayo cohort, one patient was missing data for stromal TILs; seven patients were missing Nodal Status, two patients were missing data for Ki-67; and 71 patients were missing data for luminal androgen receptor (LAR) subtype. In the Radboud cohort, 9 patients were missing stromal TILs.

### Follow-up and outcomes by cohort

**Fig. 1:**
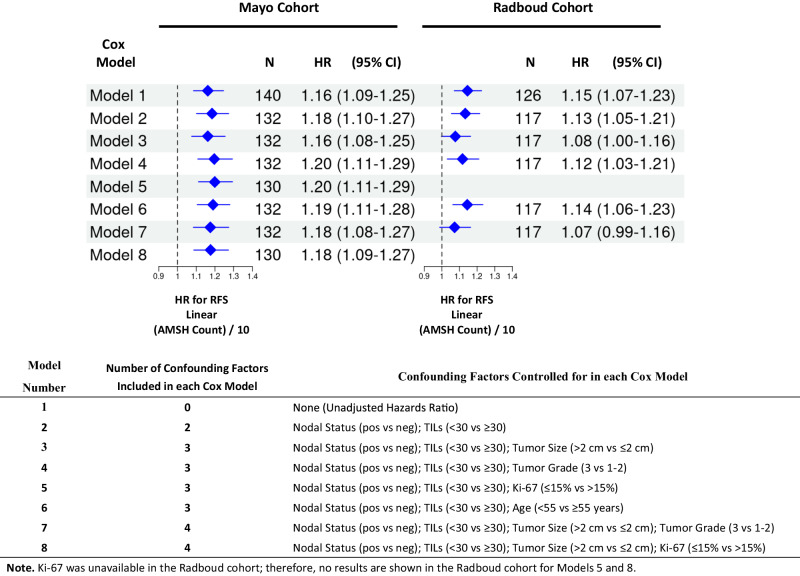
Effect of linear AMSH counts on recurrence-free survival (RFS) in each cohort. Unadjusted (Model 1) and adjusted (Models 2–8) hazard ratios (HR) and 95% confidence intervals (CIs) are shown for different sets of potential confounding factors. Each Cox model considered in the Mayo Cohort (left panel) was repeated in the Radboud Cohort (right panel) to assess consistency in the magnitude of the HR and 95% CI. Based on purposeful covariate selection, the final model chosen was Model 3.

Given that patients from the Mayo Cohort underwent surgery during a period spanning nearly three decades, we evaluated whether survival changed over time by fitting a Cox model for OS with an indicator for time interval as the only covariate (5-year increments, with 1985–1990 serving as the reference period). No significant differences were noted in survival across quinquennia (Table S1). After controlling for nodal status, tumor size, and stromal TILs, AMSH counts were independently associated with RFS (Model 3 [Final Model], Fig. 1; *P* < 0.001). In the final model (Model 3), for every 10-point increase in the AMSH count, there was a 16% increase in the risk of experiencing an RFS event (HR 1.16, 95% CI 1.08–1.25). We corroborated our findings in the Radboud Cohort (Model 3, Fig. 1). For every 10-point increase in the AMSH count, there was an 8% increase in the risk of experiencing an RFS event (HR 1.08, 95% CI 1.00–1.16). The effect of AMSH counts on RFS (HR) remained similar in the Mayo Cohort after controlling for different sets of potential confounding factors (Fig. 1), including when controlling for tumor grade (Models 4 and 7) and Ki-67 (Model 5). In the Radboud Cohort, the effect of AMSH count became attenuated after controlling for tumor size (Models 3 and 7). For instance, the HR was 1.13 after controlling for nodal status and TILs, while the HR was 1.08 after controlling for nodal status, TILs, and tumor size (Fig. 1).

In the Mayo Cohort, AMSH counts were independently associated with OS (Table S2, *P* = 0.041). After controlling for nodal status, tumor size, and stromal TILs in the final model, there was a 7% increase in the risk of death for every 10-point increase in the AMSH count (HR 1.07, 95% CI 1.00–1.14). The corresponding HR in the Radboud Cohort was similar in magnitude; however, the CI was wider and included the null value (HR 1.05, 95% CI 0.97–1.14).

**Fig. 2:**
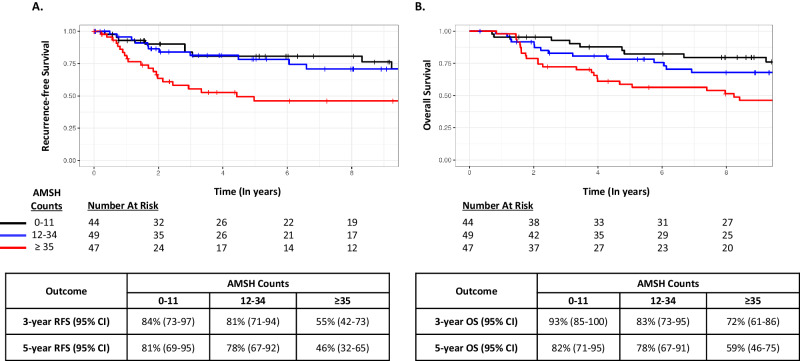
Survival outcomes according to AMSH count terciles in the Mayo Cohort. **A** Recurrence-free survival. **B** Overall survival.

**Fig. 3:**
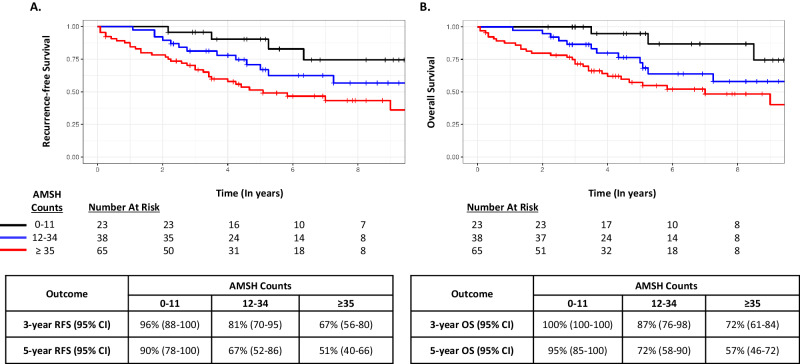
Survival outcomes according to AMSH count terciles in the Radboud Cohort. **A** Recurrence-free survival. **B** Overall survival. Analyses were based on terciles determined in the Mayo Cohort.

The unadjusted KM curves according to terciles based on the Mayo Cohort (0–11; 12–34; ≥35) and corresponding 3- and 5-year RFS and OS rates for each cohort are shown in Figs. 2 and 3. In both cohorts, patients with TNBC and AMSH counts in the lowest tercile consistently displayed the best RFS and OS, while those with AMSH in the highest tercile had the worst outcomes. These findings remained similar when restricting the analyses to patients with T1N0 tumors (Supplementary Table 3 and Supplementary Fig. 2), who are the most likely candidates for future trials evaluating systemic therapy de-escalation strategies.

**Fig. 4:**
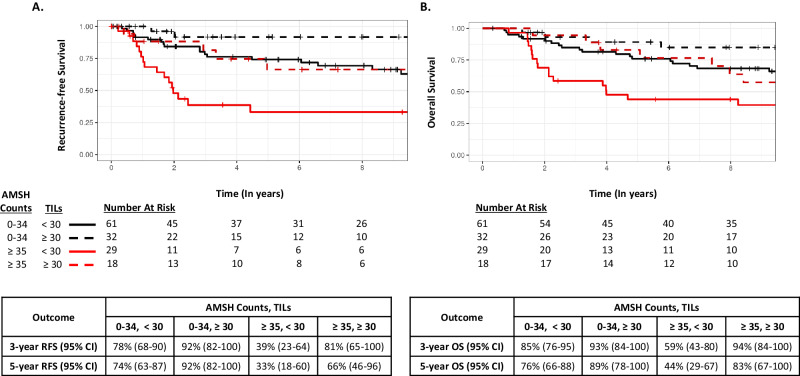
Survival outcomes according to AMSH counts (<35 vs ≥35) and TILs (<30 vs ≥30) in the Mayo Cohort. **A** Recurrence-free survival. **B** Overall survival.

**Fig. 5:**
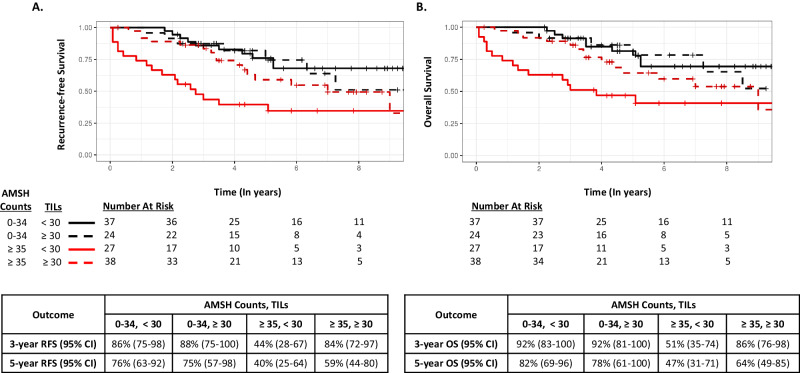
Survival outcomes according to AMSH counts (<35 vs ≥35) and TILs (<30 vs ≥30) in the Radboud Cohort. **A** Recurrence-free survival. **B** Overall survival.

To explore whether high AMSH counts (T3: ≥35) coupled with low TILs (≤30% or ≤50%) conferred worse RFS and OS, KM curves were generated according to 4 subgroups: low TILs and low AMSH counts; low TILs and high AMSH counts; high TILs and low AMSH counts; and high TILs and high AMSH counts (Figs. 4 and 5 [using a TIL threshold of 30%], and Supplementary Figure 3 [using a TIL threshold of 50%]). While the KM curves suggest a worse RFS and OS in the subgroup of patients who had low TILs and high AMSH counts within each cohort, the interaction between TILs and AMSH counts did not achieve statistical significance at any reasonable level in a multivariable-adjusted Cox model that included the factors AMSH counts (≥35; <35) and TILs (≤30; >30), and the two-way interaction between these factors (all *P* > 0.10).

## Discussion

Using two separate cohorts of patients with resected TNBC who did not receive systemic therapy, we identified that AMSH counting was strongly prognostic for RFS and OS, independent of nodal status, tumor size, and stromal TILs. In addition, despite the anticipated strong correlation between histologic grade and Ki-67 proliferation index with AMSH counts, neither tumor grade nor Ki-67 was prognostic in either cohort, suggesting that AMSH quantification may offer additional prognostic value over these two traditional proliferation biomarkers. It is important to note, however, that Ki-67 was evaluated on whole slide images (as per common clinical practice), and not per the hotspot analyses pursued in the AMSH counting method (emulating the Nottingham grading methods). As such, conclusions regarding the prognostic advantage of AMSH counts vs Ki-67 cannot be derived from this study.

Gene expression profiles (including proliferation genes) and proliferation biomarkers are commonly used in clinics to identify patients with HR-positive breast cancer who can safely avoid chemotherapy^16^. However, these approaches have not found clinical utility in TNBC. For example, the genomic grade index (GGI)—developed to reclassify tumors with intermediate histologic grade into high versus low genomic grade—was associated with recurrence risk only in HR-positive breast cancer^5^. A previous assessment of the impact of AMSH counts using the same tool applied in the current study failed to identify prognostic utility in a cohort of unselected patients with TNBC^9^. Notably, this previous evaluation included patients with TNBC who received and did not receive systemic chemotherapy. In contrast, our analysis focused exclusively on patients with early-stage TNBC who did not receive systemic therapy, allowing us to assess the natural history of the disease. We postulate that the difference in the prognostic ability of AMSH counting in systemically untreated versus treated TNBC may be partly explained by the known differential impact of cytotoxic chemotherapy on high versus low proliferating tumors. Specifically, we hypothesize that systemic therapy may disproportionally impact the natural history of TNBC with high AMSH, improving its prognosis and bringing it closer to untreated TNBC with low AMSH counts, thus obscuring the apparent value of such biomarkers in the context of systemic therapy. If validated in prospective datasets of chemotherapy versus no chemotherapy, incorporation of AMSH to other known prognostic biomarkers (i.e., age, tumor size, nodal status, TILs) may provide clinical utility in aiding the decision to administer systemic therapy or not in subsets of patients predicted to have a more favorable prognosis.

Given the substantial risk of early cancer recurrence and mortality, most patients with TNBC are recommended to receive neoadjuvant or adjuvant systemic therapy^17,18^. Recently, a neoadjuvant approach has been favored, given its potential for allowing less extensive locoregional therapy and permitting the assessment of pathologic response. However, efforts to increase the rates of pCR have led to systemic therapy approaches ever more intensive, most recently culminating in the KEYNOTE-522 regimen^19,20^, a 5-drug regimen currently recommended for most patients with stage II or III TNBC. The recommendation to pursue this intensive chemoimmunotherapy regimen is purely based on clinical stage (tumor size and nodal involvement) and does not incorporate other biomarkers. While the use of this regimen represents a significant leap in clinical TNBC management, not only improving pCR but also event-free survival, it is also associated with significant treatment-related adverse events, often longstanding. Therefore, refined stratification strategies are urgently needed to identify patients most likely to need such intensive therapy (or not). Likely, such precision-medicine approaches can primarily be accomplished by incorporating robust biomarkers that better recapitulate disease biology and identify patients at the lowest versus the highest risk of recurrence before systemic therapy is administered.

Immune-related biomarkers appear among the most promising candidates for risk stratification among patients with TNBC. Multiple studies have demonstrated that patients with “immunologically hot” TNBC (characterized by high TILs) have better outcomes than those with “immunologically cold” tumors in nearly all clinical settings, including higher pCR rates following neoadjuvant chemotherapy^21^, better survival following adjuvant chemotherapy^22^, and better survival even in the absence of systemic therapy^4,12,23^. Here, we offer an immune-agnostic biomarker focused on proliferation, which provides prognostic value independent of TILs in two separate cohorts of patients with TNBC. Intriguingly, the impact of AMSH counts on prognosis appeared to be primarily driven by the pronounced effect among patients with TNBC with low TILs. In our exploratory analysis, patients with both low TILs and high AMSH counts exhibited a particularly poor prognosis without cytotoxic chemotherapy. While numbers are small and statistical significance was not achieved, this latter finding suggests that proliferation biomarkers may be particularly relevant in immunologically cold TNBC tumors, despite not being historically helpful in unselected TNBC patients. Our findings in TNBC not treated with chemotherapy are consistent with recent studies suggesting that genomic proliferation signatures are associated with the attainment of pCR after neoadjuvant chemoimmunotherapy in TNBC tumors with low TILs^24,25^.

TNBC is an operational term that encompasses vastly heterogeneous tumors with diverse clinical outcomes and a broad spectrum of histologic and molecular features^26–33^. Of the molecular TNBC subtypes, the LAR subtype—representing ~20% of TNBCs—typically exhibits lower histologic grade and lower Ki-67 proliferation index^15^. Clinically, it has been reported to occur in older individuals, to achieve pCR less frequently, and to exhibit a pattern of recurrence resembling HR-positive breast cancers (later recurrences, more frequent bone involvement)^15,34^. Given this, we evaluated differences in AMSH counts between LAR and non-LAR TNBC in the Mayo cohort (not available in the Radboud Cohort). While the numbers are small, 60% of LAR tumors had AMSH counts in the bottom two quartiles, while 78% of non-LAR tumors had AMSH counts in the top two quartiles (Table 2). Further work is planned to evaluate gene expression differences between AMSH high versus low TNBC in the Mayo Cohort.

Our study has several strengths, including the robustness of the convolutional neural network tool trained on PHH3 staining, which offers a contrast-rich stain with high reproducibility and does not stain apoptotic cells—often confused for mitotic cells. In addition, our methodology offers the advantage of leveraging universally available H&E-stained slides without requiring manual annotation. Our findings were consistent in two separate cohorts of TNBC patients, explicitly focusing on patients who did not receive systemic therapy (a rare subset in the current era and allowing evaluation of the natural history of TNBC). Furthermore, the prognostic implications of AMSH counts were independent of other established prognostic biomarkers, including age, nodal status, tumor size, and TILs. Our study also has important limitations, including its retrospective nature, the relatively small size of the two cohorts, and the unavailability of Ki-67 in the Radboud cohort. Pertaining to the AMSH count evaluation method, pre-analytical variables such as differences in tissue sectioning, staining, and digitation can affect its performance, which may partly explain the differences in counts observed between the two cohorts. Furthermore, the predictive value of this tool in the context of neoadjuvant systemic therapy (and whether it can predict the achievement of pCR) remains unknown. To overcome these limitations, work optimizing the algorithm to make it less susceptible to pre-analytical variables is ongoing. To further validate our findings, evaluation of AMSH counts in additional cohorts should be pursued, including larger cohorts of patients with systemically untreated TNBC, patients treated in the neoadjuvant setting, and prospective retrospective analyses of clinical trial datasets. If further validated, AMSH counting should be prospectively evaluated in systemic therapy de-escalation clinical trials.

## Methods

### AMSH count detection from digitized whole slide images

**Fig. 6:**
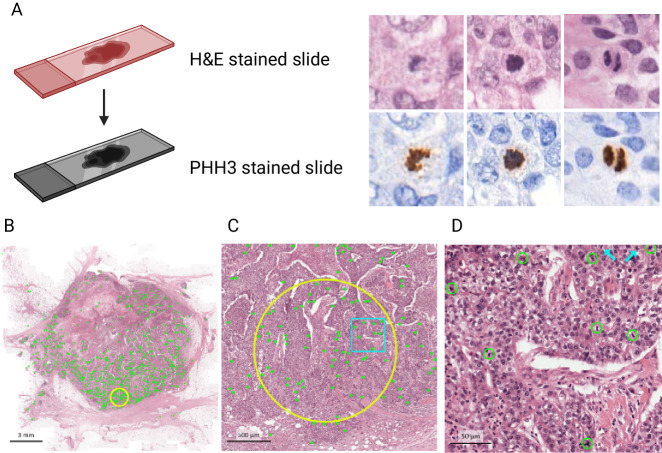
Automatic Mitotic Spindle Hotspot counts workflow. **A** Destained hematoxylin & eosin (H&E) slides are subsequently restained with phosphohistone-H3 (PHH3), which provides a rich contrast image without staining apoptotic figures. This image is subsequently registered to traditional H&E to allow mitosis detection independently of manual annotation. **B** Every detected mitotic figure is represented by a green dot. A 2 mm^2^ area with the highest density of mitotic figures is designated as a “hotspot” and circled in yellow. **C** The hotspot area as found by the deep learning algorithm at higher magnification. The blue rectangle within the yellow hotspot circle is magnified in **D**. **D** Mitotic figures found by the deep learning algorithm are circled in green. Two mitotic figures were missed by the algorithm (blue arrows, top right). Image modified from Tellez et al.^35^ and Balkenhol et al.^9^ and designed in part using Biorender.com.

To determine AMSH counts, we used a state-of-the-art convolutional neural network trained to detect mitoses in digitized whole slide images. Details of the algorithm development, training, and validation were published previously^8,35^ and illustrated in Fig. 6. Briefly, hematoxylin and eosin (H&E) slides were scanned, destained, and subsequently restained with phosphohistone-H3 (PHH3)—a protein involved in chromatin condensation and decondensation present in the G2-M cell cycle transition^36–39^. The automatic analysis of mitotic activity using PHH3 and registering it to H&E allowed us to generate training data for mitosis detection in H&E whole slide images in a scalable and reproducible manner that was independent of manual annotation. This method first identifies all mitotic spindles in the entire whole slide image, and subsequently automatically identifies a circular region of 2 mm^2^ with the highest mitotic density (i.e., hotspots).

### Mayo Clinic Cohort evaluation

We obtained AMSH counts in tumors from a cohort of patients with operable TNBC from Mayo Clinic and focused on patients who received locoregional but no systemic therapy. Eligibility criteria for inclusion, patient characteristics, and methods of pathology assessment (e.g., Ki-67 and TILs) of the Mayo TNBC cohort have been previously published^4^. Briefly, available formalin-fixed, paraffin-embedded tumors from patients who underwent upfront surgery (no neoadjuvant therapy) for stage I-III breast cancer, clinically determined to be HER2 not amplified or unknown, between 1 January 1985 and 31 December 2012 at Mayo Clinic were further evaluated pathologically. Tumors were centrally evaluated for ER, PR, and HER2 per the 2013 American Society of Clinical Oncology/College of American Pathologists guidelines^40^. For centrally confirmed TNBC tumors, we abstracted clinical data and digitally assessed the Ki-67 labeling index (MIB-1 monoclonal antibody, Dako, Carpinteria, CA, 1:400) using whole slide images and not based on hotspot analysis. Slides from the Mayo Cohort were digitally scanned at 40× using a Leica Aperio scanner, models GT450 or AT2. Slides from the Radboud cohort were digitally scanned at a spatial resolution of 0.25 μm/pixel using a Pannoramic 250 Flash II slide scanner (3DHistech, Hungary). A dedicated breast pathologist blinded to clinical information quantified TILs on full-face H&E sections from the surgical specimen, following the TILs Working Group recommendations^41^. From 605 eligible patients with centrally confirmed TNBC and clinical outcomes data, 182 patients underwent surgery and received no subsequent adjuvant systemic therapy and are the focus of these analyses.

### Radboud University Medical Center Cohort evaluation

After evaluating whether AMSH counts were prognostic in the Mayo cohort, we sought to corroborate our findings in a separate cohort of systemically untreated TNBC patients from Radboud University Medical Center in the Netherlands (Radboud Cohort). Eligibility criteria for inclusion in this cohort are detailed elsewhere^42^. Briefly, the Netherlands Comprehensive Cancer Registry was leveraged to identify patients diagnosed with non-metastatic TNBC between 2006 through 2014, and who were treated with upfront surgery. Patients who received neoadjuvant therapy were excluded. Of 597 patients with TNBC meeting eligibility criteria for this cohort, AMSH counts were obtained in half of them (*n* = 298). For these analyses, we focused on 126 patients who had not received subsequent adjuvant systemic therapy and who did not have adenoid cystic carcinoma.

### Statistical analyses

Analyses were conducted first in the Mayo Clinic Cohort. The Cochran-Armitage test for trend^43^ was applied to test for an association between a dichotomized baseline factor and increasing AMSH counts categorized by terciles; baseline factors included age (<55; ≥55), menopausal status (premenopausal; postmenopausal), tumor size (≤2 cm; >2 cm), nodal status (negative; positive), histologic grade (1–2; 3), TILs (>30%; ≤30%), TILs (≥50%; <50%), Ki-67 proliferation index (≤15%; >15%), and LAR subtype (Non-LAR; LAR). Without a known functional form of AMSH counts to include in a multivariable proportional hazards model, fractional polynomial analysis^44^ with a multivariable proportional hazards regression model that included nodal status and TILs was applied to determine whether AMSH counts were prognostic of RFS and its functional form. AMSH counts were rescaled by dividing by 10 before being power-transformed. In the closed test procedure^45^, the linear model was compared to the best two-term model. Because this test did not achieve statistical significance at any reasonable level (*P* = 0.20), we had reasonable evidence to assume that the log hazard was linear in AMSH counts. Two graphical methods were applied to confirm the assumption of linearity in the log hazard for the continuous AMSH counts. The graphical methods included the quartile design variable method (i.e., the estimated log hazard ratios for the design variables were plotted versus the midpoints of the intervals defined by the cut-points) and the smoothed added variable plot^46^. Sensitivity analyses were performed to ensure the linear relationship was not influenced by large AMSH counts. We applied purposeful selection of covariates, which considered issues of confounding, effect modification, and overfitting, and thoroughly evaluated the model for assumptions, influential observations, and tests for goodness-of-fit. Covariates considered for inclusion comprised nodal status, TILs, tumor size, histological grade, menopausal status, age, and Ki-67 proliferative index. The final model included the linear AMSH counts (rescaled by 10), nodal status (positive vs. negative), stromal TILs (<30 vs. ≥30), and tumor size (≤2 vs. >2 cm). The same final model resulted for OS.

We report the adjusted hazards ratio for AMSH counts (scaled by 10 corresponding to a 10-unit change) and 95% confidence interval (CI) from the final model for both RFS and OS. Additionally, AMSH counts were categorized according to terciles (0–11; 12–34; ≥35), and Kaplan–Meier (KM) curves were derived for both RFS and OS. We report 3- and 5-year RFS and OS percentages and corresponding 95% CIs.

Following analyses conducted in the Mayo Cohort, the same fractional polynomial analysis was applied to the Radboud Cohort to confirm the linearity assumption in the log hazard for the AMSH counts for both outcomes. We report the adjusted hazards ratio for AMSH counts (scaled by 10) and 95% CI from the respective final models for RFS and OS identified in the Mayo Cohort. Additionally, we calculated the RFS and OS KM curves according to the same tercile thresholds based on the Mayo Cohort.

For RFS and OS within each cohort, we explored all Cox models that included AMSH count, one of the baseline factors, and the two-way interaction between that factor and AMSH count. Our goal was to ascertain if there were factors for which the magnitude of the effect of AMSH count differed according to the level of the factor. Given the lack of evidence that a baseline factor modified the effect of AMSH counts in our current datasets (data not shown), we assessed whether certain baseline factors confounded the effect of AMSH counts on RFS and OS. Eight Cox models (Fig. 1), including the model without adjustment (Model 1) and the final model selected based on purposeful selection (Model 3), were considered. Our goal was to evaluate whether the magnitude of the hazard ratio for AMSH count (scaled by 10) changed in any material way after adjusting for different sets of baseline factors. Due to the risk of overfitting, the maximum number of baseline factors considered in a given Cox model was four. We present a side-by-side forest plot for RFS to show the magnitude of the hazards ratio for AMSH count across each model for both cohorts (Fig. 1).

Survival outcomes (RFS and OS) were defined according to the Standardized Definitions for Efficacy EndPoints (STEEP) in Adjuvant Breast Cancer Clinical Trials, second edition^47^. Results in the Mayo and Radboud Cohorts are reported at a median follow-up of 8 and 5 years, respectively. All analyses were performed using R Statistical Software (v4.1.2; R Core Team 2021). Fractional polynomial analysis was performed via the *mfp* R package (Ambler G. and Benner A. 2022). *P* values are two-sided and are reported as a continuous measure of evidence against the null. No adjustment was made for performing multiple tests.

### Ethical consideration**s**

The study conformed to Health Insurance Portability and Accountability Act (HIPPA) guidelines and was approved by the respective Institutional Review Boards (IRB) at Mayo Clinic and Radboud University Medical Center. The IRBs waived the requirements for patient informed consent due to the retrospective and non-interventional nature of this study. This study complied with all relevant ethical regulations, including the Declaration of Helsinki.

### Reporting summary

Further information on research design is available in the Nature Research Reporting Summary linked to this article.

### Supplementary information

Supplementary Material

Reporting Summary

## Data Availability

The data that support the findings of this study are available from the corresponding author upon reasonable request.
