# Supplementary material for: Automated mitotic spindle hotspot counts are highly associated with clinical outcomes in systemically untreated early-stage triple-negative breast cancer
Source: NPJ Breast Cancer. 2024 Mar 29;10:25. doi: 10.1038/s41523-024-00629-3 (PMC10980681; doi:10.1038/s41523-024-00629-3)
Supplement: Supplementary file 1 — Supplementary Material [file 41523_2024_629_MOESM1_ESM.pdf]

Supplementary material for "Automated mitotic spindle hotspot counts are highly associated with clinical outcomes in systemically untreated early-stage triple-negative breast cancer"

Table of Contents

Supplementary Figure 1. Distribution of AMSH counts
Supplementary Table 1. Overall survival across quinquennia in the Mayo Cohort
Supplementary Table 2. Estimated Hazards Ratio and 95% Confidence Intervals from Selected Model (Model 3) on Overall Survival
Supplementary Table 3. Effect of linear AMSH counts on Recurrence-Free Survival within the T1N0 subgroup, unadjusted and adjusted for TILs.
Supplementary Figure 2. Clinical outcomes according to AMSH counts in T1N0 TNBC
Supplementary Figure 3. Clinical outcomes according to AMSH counts and TILs (using a 50% threshold) in the Mayo and Radboud Cohorts

---

Supplementary Figure 1: Distribution of AMSH counts

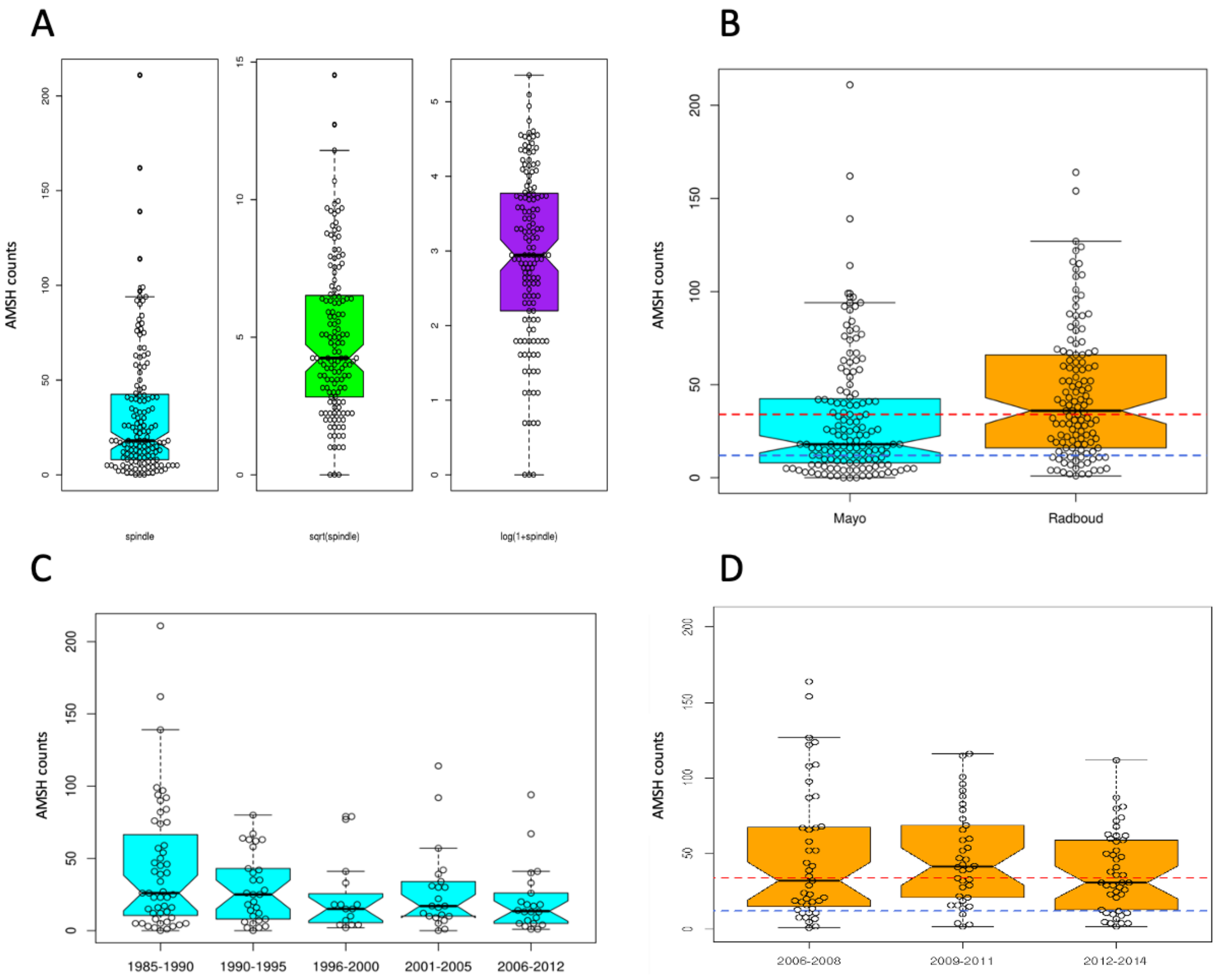

Supplementary Figure 1. Panel A. Boxplots of AMSH counts (original scale [left]; square root scale [middle] for the Mayo Cohort; natural logarithm scale [right]). **Panel B** shows the distribution of AMSH counts in each cohort (original scale). **Panel C** shows the distribution of AMSH within each 5-year increment (1985-1990, 1990-1995, 1996-2000, 2001-2005, 2006-2012) for the Mayo Cohort. **Panel D** shows the distribution within each 2-year increments for the Radboud cohort (2006-2008, 2009-2011, 2012-2014). We did not observe clinically important distributional differences over time.

Supplementary Table 1: Overall survival across quinquennia in the Mayo Cohort.

Year of Surgery	P value
1985-1990	(Reference)
1991-1995	P = 0.64
1996-2000	P = 0.22
2001-2005	P = 0.15
2006-2012	P = 0.11

Given that patients in the Mayo Cohort were treated with surgery between 1985 and 2012 (nearly three decades), there is potential for changes in survival rates due to changes in approaches to diagnosis and management of breast cancer, or other unaccounted factors. To evaluate whether there was a drift in survival over time in the Mayo Cohort, we fit a Cox model for overall survival using indicators for time intervals as the only covariates with 1985-1990 serving as the reference category. There were no statistically significant differences in overall survival in any time period compared to the reference period of 1985-1990.

Supplementary Table 2. Estimated Hazards Ratio and 95% Confidence Intervals from Selected Model (Model 3) on Overall Survival for the Mayo Cohort

Variable	Hazards Ratio	95% CI	P
Nodal Status (positive vs negative)	4.29	(2.29, 8.02)	<0.0001
Stromal TILs (≥30 vs <30)	0.44	(0.24, 0.78)	0.006
(AMSH Count) / 10 (Linear)	1.07	(1.00, 1.14)	0.041
Tumor Size (>2cm vs ≤ 2 cm)	1.81	(1.01, 3.21)	0.045

Supplementary Table 3. Effect of linear AMSH count on recurrence-free survival within the T1N0 subgroup, unadjusted and adjusted for TILs.

Cox Model T1N0 Subgroup	Mayo Cohort (N = 87) Linear (AMSH Count) / 10			Radboud Cohort (N = 70) Linear (AMSH Count) / 10		
	Hazards Ratio	95% CI	P	Hazards Ratio	95% CI	P
Unadjusted	1.21	1.03, 1.43	0.024	1.19	1.02, 1.38	0.024
Adjusted for TILs (<30; ≥30)	1.24	1.04, 1.46	0.014	1.19	1.00, 1.43	0.052

Supplementary Figure 2. Clinical outcomes according to AMSH counts in T1N0 TNBC

A

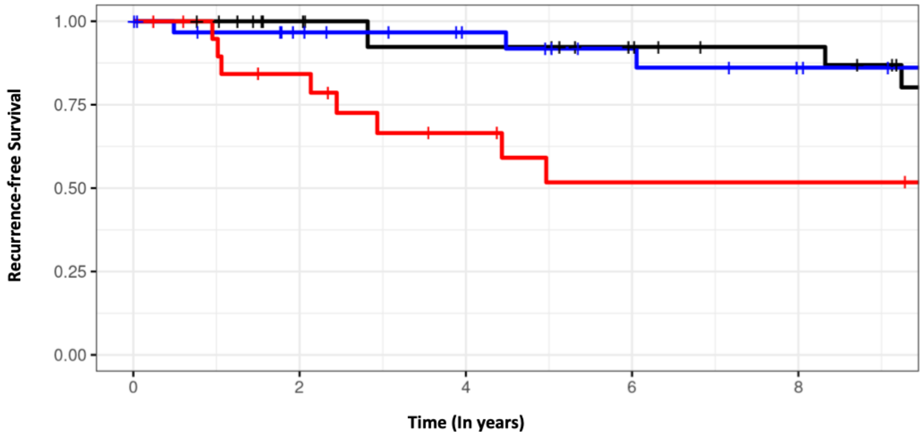

AMSH Counts	Number at Risk				
0-11	34	28	24	20	17
12-34	32	25	20	16	13
≥ 35	21	15	10	7	7

Outcome	AMSH Counts		
	0-11	12-34	≥35
3-year RFS (95% CI)	92% (83-100)	97% (91-100)	67% (48-93)
5-year RFS (95% CI)	92% (83-100)	92% (81-100)	52% (32-84)

B

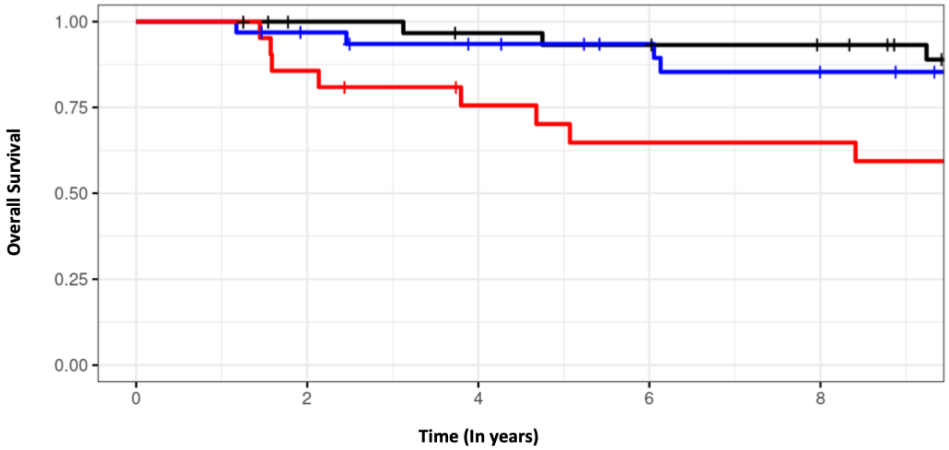

AMSH Counts	Number at Risk				
0-11	34	30	28	27	25
12-34	32	29	26	23	20
≥ 35	21	18	14	12	12

Outcome	AMSH Counts		
	0-11	12-34	≥35
3-year RFS (95% CI)	95% (86-100)	82% (68-100)	77% (62-95)
5-year RFS (95% CI)	95% (86-100)	70% (51-94)	64% (47-86)

C

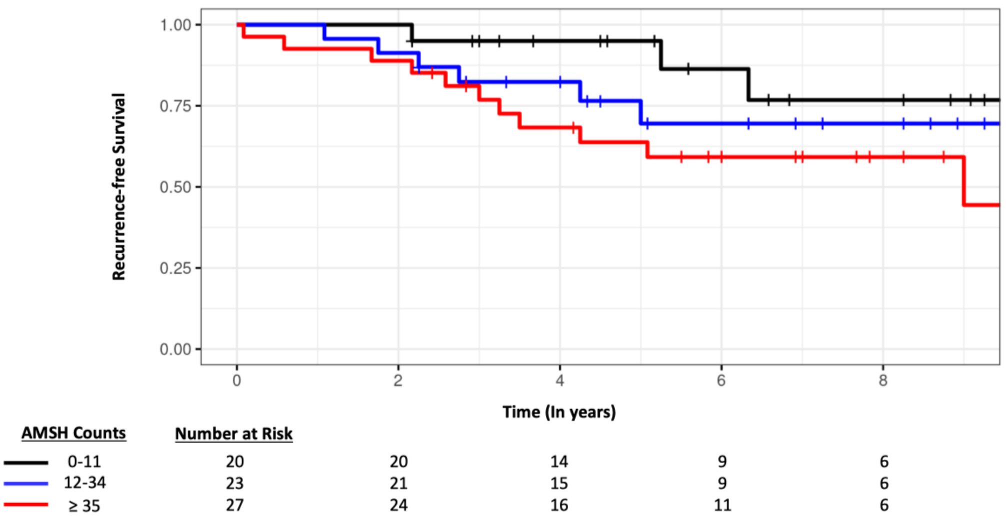

Outcome	AMSH Counts		
	0-11	12-34	≥35
3-year RFS (95% CI)	95% (86-100)	82% (68-100)	77% (62-95)
5-year RFS (95% CI)	95% (86-100)	70% (51-94)	64% (47-86)

D

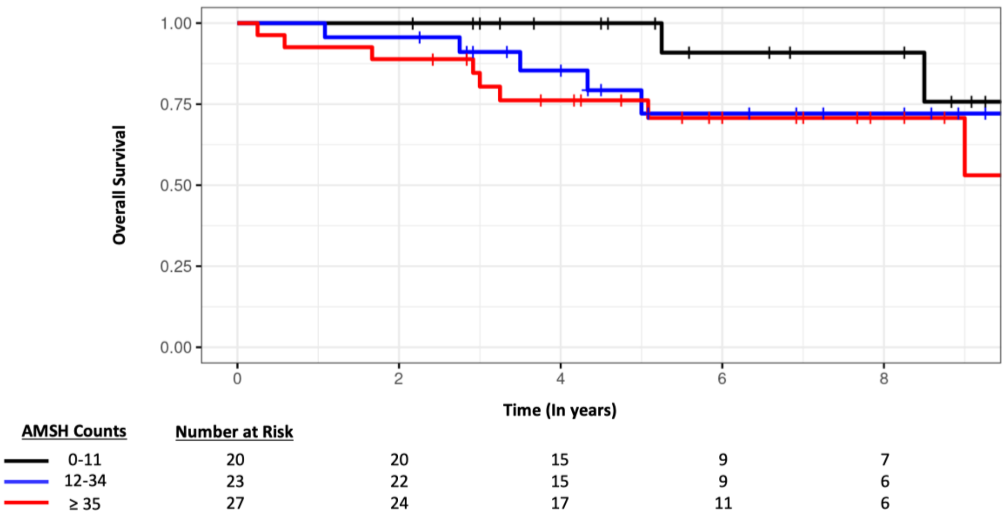

Outcome	AMSH Counts		
	0-11	12-34	≥35
3-year OS (95% CI)	100% (100-100)	91% (80-100)	80% (66-98)
5-year OS (95% CI)	100% (100-100)	72% (54-97)	76% (61-95)

Supplementary Figure 2. Panel A: RFS in the Mayo Cohort (T1N0) according to AMSH terciles, **Panel B:** OS in the Mayo Cohort (T1N0) according to AMSH terciles, **Panel C:** RFS in the Radboud Cohort (T1N0) according to AMSH terciles as determined in the Mayo Cohort, **Panel D:** OS in the Radboud Cohort (T1N0) according to AMSH terciles as determined in the Mayo Cohort

Supplementary Figure 3. Clinical outcomes according to AMSH counts and TILs (using a 50% threshold) in the Mayo and Radboud Cohorts. **Panel A:** RFS in the Mayo Cohort, **Panel B:** OS in the Mayo Cohort, **Panel C:** RFS in the Radboud Cohort, **Panel D:** OS in the Radboud Cohort.

A

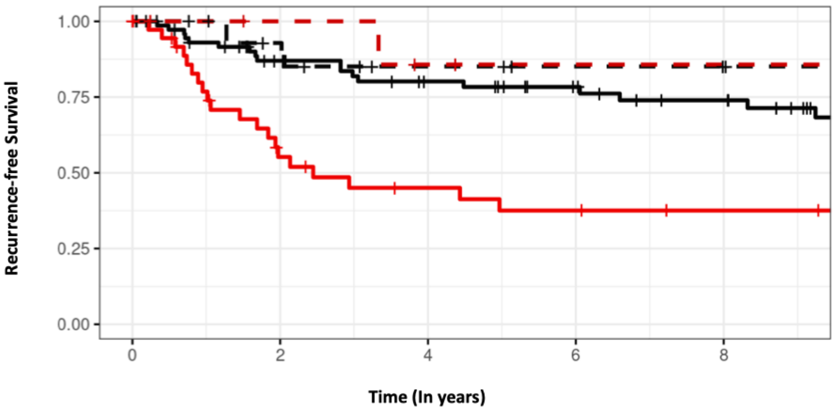

AMSH Counts	TILs	Number at Risk				
0-34	< 50	74	55	44	37	31
0-34	≥ 50	19	12	8	6	5
≥ 35	< 50	38	17	12	10	8
≥ 35	≥ 50	9	7	5	4	4

Outcome	AMSH Counts, TILs			
	0-34, < 50	0-34, ≥ 50	≥ 35, < 50	≥ 35, ≥ 50
3-year RFS (95% CI)	82% (73-92)	85% (68-100)	45% (31-66)	100% (100-100)
5-year RFS (95% CI)	78% (69-89)	85% (68-100)	38% (24-60)	86% (63-100)

B

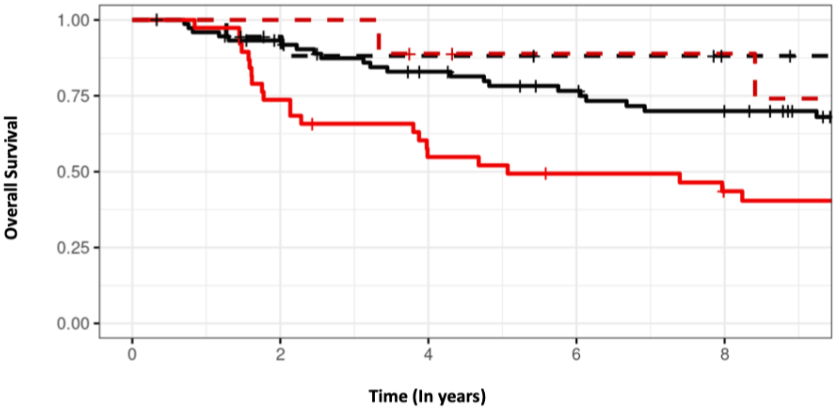

AMSH Counts	TILs	Number at Risk				
0-34	< 50	74	65	54	47	41
0-34	≥ 50	19	15	14	13	11
≥ 35	< 50	38	28	20	17	14
≥ 35	≥ 50	9	9	7	6	6

Outcome	AMSH Counts, TILs			
	0-34, < 50	0-34, ≥ 50	≥ 35, < 50	≥ 35, ≥ 50
3-year RFS (95% CI)	87% (80-96)	88% (74-100)	66% (52-83)	100% (100-100)
5-year RFS (95% CI)	78% (69-89)	88% (74-100)	52% (38-71)	89% (71-100)

C

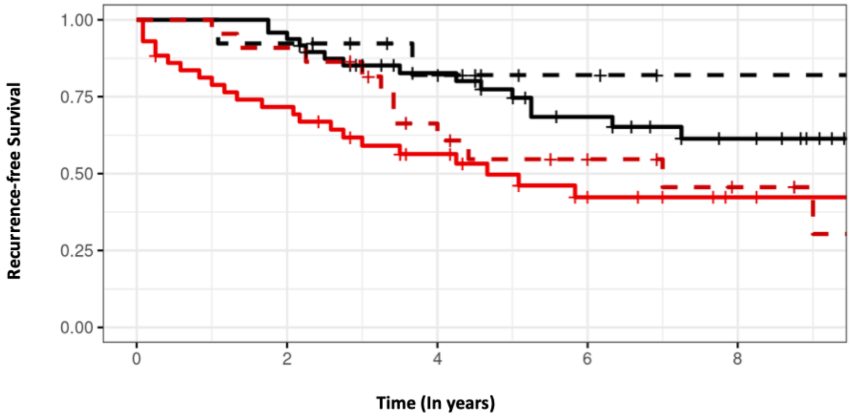

AMSH Counts	TILs		Number at Risk				
0-34	< 50	———	48	46	32	21	14
0-34	≥ 50	– – –	13	12	8	3	1
≥ 35	< 50	———	43	30	19	10	4
≥ 35	≥ 50	– – –	22	20	12	8	4

Outcome	AMSH Counts, TILs			
	0-34, < 50	0-34, ≥ 50	≥ 35, < 50	≥ 35, ≥ 50
3-year RFS (95% CI)	85% (76-96)	92% (79-100)	59% (46-76)	82% (67-100)
5-year RFS (95% CI)	75% (63-89)	82% (62-100)	50% (36-82)	55% (36-82)

D

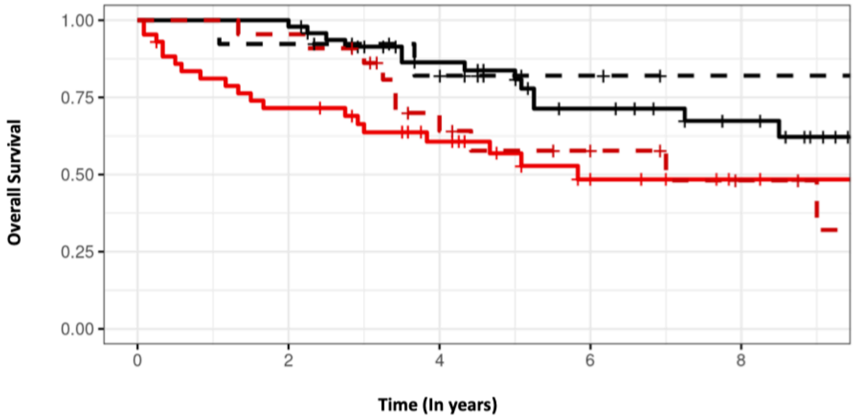

AMSH Counts	TILs		Number at Risk				
0-34	< 50	———	48	48	33	21	15
0-34	≥ 50	– – –	13	12	8	3	1
≥ 35	< 50	———	43	30	20	10	4
≥ 35	≥ 50	– – –	22	21	12	8	4

Outcome	AMSH Counts, TILs			
	0-34, < 50	0-34, ≥ 50	≥ 35, < 50	≥ 35, ≥ 50
3-year RFS (95% CI)	91% (84-100)	92% (79-100)	64% (51-80)	86% (73-100)
5-year RFS (95% CI)	81% (70-94)	82% (62-100)	57% (43-75)	58% (39-86)
