# Supplementary material for: Automated mitotic spindle hotspot counts are highly associated with clinical outcomes in systemically untreated early-stage triple-negative breast cancer
Source: NPJ Breast Cancer. 2024 Mar 29;10:25. doi: 10.1038/s41523-024-00629-3 (PMC10980681; doi:10.1038/s41523-024-00629-3)
Supplement: Supplementary file 2 — Reporting Summary [file 41523_2024_629_MOESM2_ESM.pdf]

nature portfolio

Corresponding author(s): Roberto Leon-Ferre

Last updated by author(s): Nov 23, 2023

Reporting Summary

Nature Portfolio wishes to improve the reproducibility of the work that we publish. This form provides structure for consistency and transparency in reporting. For further information on Nature Portfolio policies, see our Editorial Policies and the Editorial Policy Checklist.

Statistics

For all statistical analyses, confirm that the following items are present in the figure legend, table legend, main text, or Methods section.

n/a	Confirmed	
☐	☒	The exact sample size (*n*) for each experimental group/condition, given as a discrete number and unit of measurement
☐	☒	A statement on whether measurements were taken from distinct samples or whether the same sample was measured repeatedly
☐	☒	The statistical test(s) used AND whether they are one- or two-sided<br>*Only common tests should be described solely by name; describe more complex techniques in the Methods section.*
☐	☒	A description of all covariates tested
☐	☒	A description of any assumptions or corrections, such as tests of normality and adjustment for multiple comparisons
☐	☒	A full description of the statistical parameters including central tendency (e.g. means) or other basic estimates (e.g. regression coefficient) AND variation (e.g. standard deviation) or associated estimates of uncertainty (e.g. confidence intervals)
☐	☒	For null hypothesis testing, the test statistic (e.g. *F*, *t*, *r*) with confidence intervals, effect sizes, degrees of freedom and *P* value noted<br>*Give P values as exact values whenever suitable.*
☒	☐	For Bayesian analysis, information on the choice of priors and Markov chain Monte Carlo settings
☐	☒	For hierarchical and complex designs, identification of the appropriate level for tests and full reporting of outcomes
☐	☒	Estimates of effect sizes (e.g. Cohen's *d*, Pearson's *r*), indicating how they were calculated

Our web collection on statistics for biologists contains articles on many of the points above.

Software and code

Policy information about availability of computer code

Data collection	Microsoft Excel 365
Data analysis	R

For manuscripts utilizing custom algorithms or software that are central to the research but not yet described in published literature, software must be made available to editors and reviewers. We strongly encourage code deposition in a community repository (e.g. GitHub). See the Nature Portfolio guidelines for submitting code & software for further information.

Data

Policy information about availability of data

All manuscripts must include a data availability statement. This statement should provide the following information, where applicable:

- Accession codes, unique identifiers, or web links for publicly available datasets
- A description of any restrictions on data availability
- For clinical datasets or third party data, please ensure that the statement adheres to our policy

Upon publication, the data underlying this article will be available in the Synapse repository (synapse.org) SynID: syn51424934.

Research involving human participants, their data, or biological material

Policy information about studies with human participants or human data. See also policy information about sex, gender (identity/presentation), and sexual orientation and race, ethnicity and racism.

Reporting on sex and gender	Data in this study applies to female sex only, as it is focused on triple negative breast cancer. While there was no exclusion of male subjects, the vast majority of breast cancer occurring in males is estrogen receptor positive. There were no men with triple negative breast cancer in either cohort
Reporting on race, ethnicity, or other socially relevant groupings	Race and ethnicity were not available in either cohort
Population characteristics	A total of 182 patients in the Mayo cohort and 130 patients in the Radboud cohort were treated with locoregional therapy but no adjuvant or neoadjuvant systemic therapy. Patient characteristics shown in Table 1
Recruitment	Cases were recruited retrospectively, by reviewing all cases of triple negative breast cancer in each institution that met the criteria.
Ethics oversight	Mayo Clinic and Radboud University Medical Center

Note that full information on the approval of the study protocol must also be provided in the manuscript.

Field-specific reporting

Please select the one below that is the best fit for your research. If you are not sure, read the appropriate sections before making your selection.

☒ Life sciences ☐ Behavioural & social sciences ☐ Ecological, evolutionary & environmental sciences

For a reference copy of the document with all sections, see nature.com/documents/nr-reporting-summary-flat.pdf

Life sciences study design

All studies must disclose on these points even when the disclosure is negative.

Sample size	182 patients in the Mayo cohort and 130 patients in the Radboud Cohort. Sample size was based on the number of patients available in each institution meeting the study criteria.
Data exclusions	This study excluded patients with triple negative breast cancer who received chemotherapy, as the focus was on patients treated with surgery and/or radiation only
Replication	Data obtained in the Mayo Clinic cohort was replicated in a separate cohort from the Radboud Medical Center
Randomization	No randomization applicable to this study, as this was not an interventional study.
Blinding	A dedicated breast pathologist blinded to clinical data quantified Tumor infiltrating lymphocytes. Otherwise, no additional blinding was applicable to this study, as it was not an interventional study

Reporting for specific materials, systems and methods

We require information from authors about some types of materials, experimental systems and methods used in many studies. Here, indicate whether each material, system or method listed is relevant to your study. If you are not sure if a list item applies to your research, read the appropriate section before selecting a response.

Materials & experimental systems

n/a	Involved in the study	
☐	☒	Antibodies
☒	☐	Eukaryotic cell lines
☒	☐	Palaeontology and archaeology
☒	☐	Animals and other organisms
☐	☒	Clinical data
☒	☐	Dual use research of concern
☐	☐	Plants

Methods

n/a	Involved in the study	
☒	☐	ChIP-seq
☒	☐	Flow cytometry
☒	☐	MRI-based neuroimaging

Antibodies

Antibodies used	For Ki-67: MIB-1 monoclonal antibody, Dako, Carpinteria, CA, 1:400
Validation	Clinically available test. Details in manuscriot

Clinical data

Policy information about clinical studies

All manuscripts should comply with the ICMJE guidelines for publication of clinical research and a completed CONSORT checklist must be included with all submissions.

Clinical trial registration	N/A
Study protocol	Not a clinical trial
Data collection	Data collection occured at Mayo Clinic and Radboud Medical center, as indicated in manuscript
Outcomes	Recurrence Free Survival and Overall survival, using STEEP 2.0 criteria

Plants

Seed stocks	NA
Novel plant genotypes	NA
Authentication	NA
